# Innate Immunity in multiple sclerosis white matter lesions: expression of natural cytotoxicity triggering receptor 1 (NCR1)

**DOI:** 10.1186/1742-2094-9-1

**Published:** 2012-01-02

**Authors:** Pascal F Durrenberger, Anna Ettorre, Fatemah Kamel, Louise V Webb, Malcolm Sim, Richard S Nicholas, Omar Malik, Richard Reynolds, Rosemary J Boyton, Daniel M Altmann

**Affiliations:** 1 Department of Medicine, Section of Infectious Diseases and Immunity, Commonwealth Building, Hammersmith Campus, Imperial College London, UK; 2 Department of Cellular & Molecular Neuroscience, Charing Cross Hospital, Charing Cross Campus, NHS Imperial College, London, UK; 3 Department of Medicine, Centre for Neuroscience, Burlington Danes, Hammersmith Campus, Imperial College London, UK

**Keywords:** Autoimmune diseases, neurodegeneration, natural killer cell, astrocyte, neuroinflammation

## Abstract

**Background:**

Pathogenic or regulatory effects of natural killer (NK) cells are implicated in many autoimmune diseases, but evidence in multiple sclerosis (MS) and its murine models remains equivocal. In an effort to illuminate this, we have here analysed expression of the prototypic NK cell marker, NCR1 (natural cytotoxicity triggering receptor; NKp46; CD335), an activating receptor expressed by virtually all NK cells and therefore considered a pan-marker for NK cells. The only definitive ligand of NCR1 is influenza haemagglutinin, though there are believed to be others. In this study, we investigated whether there were differences in NCR1^+ ^cells in the peripheral blood of MS patients and whether NCR1^+ ^cells are present in white matter lesions.

**Results:**

We first investigated the expression of NCR1 on peripheral blood mononuclear cells and found no significant difference between healthy controls and MS patients. We then investigated mRNA levels in central nervous system (CNS) tissue from MS patients: NCR1 transcripts were increased more than 5 times in active disease lesions. However when we performed immunohistochemical staining of this tissue, few NCR1^+ ^NK cells were identified. Rather, the major part of NCR1 expression was localised to astrocytes, and was considerably more pronounced in MS patients than controls. In order to further validate *de novo *expression of NCR1 in astrocytes, we used an *in vitro *staining of the human astrocytoma U251 cell line grown to model whether cell stress could be associated with expression of NCR1. We found up-regulation of NCR1 expression in U251 cells at both the mRNA and protein levels.

**Conclusions:**

The data presented here show very limited expression of NCR1^+ ^NK cells in MS lesions, the majority of NCR1 expression being accounted for by expression on astrocytes. This is compatible with a role of this cell-type and NCR1 ligand/receptor interactions in the innate immune response in the CNS in MS patients. This is the first report of NCR1 expression on astrocytes in MS tissue: it will now be important to unravel the nature of cellular interactions and signalling mediated through innate receptor expression on astrocytes.

## Background

### Natural cytotoxicity receptors

NCR1 (natural cytotoxicity triggering receptor; NKp46; CD335) is a key receptor initiating NK cell mediated cytolysis [1]. It is expressed on all human NK cells irrespective of their state of maturation and activation and has been regarded as the prototypic, pan-NK cell marker [2]. The direct killing of a target by NK cells is orchestrated by activating receptors including CD16, CD80, NCR2 (NKp44 or CD336), NCR3 (NKp30 or CD337), NKG2D (CD314), 2B4 (CD244), the novel NKp80 (KLRF1) and the killer cell immunoglobulin-like receptors-KIRs [3].

NCR1 was first identified in 1997 [4] and cloned one year later [5]. NCR1 is a 46 kDa type I transmembrane glycoprotein, characterised by two C2-type immunoglobulin-like domains in the extracellular portion and hence a member of the immunoglobulin superfamily (IgSF). NCR1 (or NKp46) shares similarities with NKp30, while NKp44 is different and is only expressed on activated NK cells [6]. The crystal structure of NCR1 shows structural similarities to LIR1, KIR2DL2, FcγRIIb and other Fc receptors [7]. Upon activation, NCR1 increases cytotoxicity, Ca^2+ ^mobilisation and cytokine production in NK cells [4]. NCR1 is not uniformly expressed, the surface density on NK cells varying between individuals. In a control population, < 20% donors display the NCR^dull ^phenotype while most donors express a high density of NCRs on NK cells, NCR^bright ^phenotype [8]. This expression difference underpins a relationship between NCR density and NK mediated-cytolytic activity [3]. Decreased NCR (NKp30 and NKp46) expression on NK cells in the elderly has been reported, potentially impacting on susceptibility to infectious, inflammatory, and neoplastic diseases [9]. Relatively little is known about NCR1 ligands. To date, the only unequivocally identified ligands for NCR1 are influenza haemagglutinin [1,10].

### NCR1 in disease

Activating NK receptors recognise stress-induced ligands and viral products. Following influenza virus infection, an increased recognition and binding of NK cells with infected cells via the NCR1 receptor is observed [11]. It has been suggested that NCR1^+ ^NK cells may have a role in mediating the pathogenesis of Crohn's disease by producing interferon-γ [12]. Furthermore, NCR1 was shown to be essential for the development of diabetes [13]. The role of NK cells in general and NCR1^+ ^cells in particular in MS is unclear. The fact that there are NK cell subsets showing varying cytokine profiles and cytotoxicity underpins uncertainty in the MS literature as to whether NK cells are pathogenic or regulatory [14]. Evidence from the murine model, experimental autoimmune encephalomyelitis (EAE) has been used to argue both views [15,16]. *Ex vivo *evidence suggests that NK cells have the capacity to lyse cultured primary oligodendrocytes and foetal astrocytes but not adult astrocytes, neurones or microglia via NKG2D ligands which are expressed in the MS brain [17]. Another study has shown NK cells to be cytotoxic to resting but not activated microglia, via NKG2D and NKp46 [18]. Evidence from EAE suggests that NK cells may be protective through their ability to make neurotrophic growth factors [19]. Evidence from humans also suggests a protective role for NK cells in MS: amelioration of MS by treatment with interferon-β [20] and anti-CD25 antibodies [21] or during pregnancy, correlates with expansion of presumed regulatory NK cells [22]. Also, CD95 (Fas) positive NK cells expand during remission [23] and are proposed to kill activated T cells. Nevertheless, studies using untreated MS patients have mostly detected deficits in NK cells function rather than differences in overall numbers between MS and controls. For instance, a reduction of an NK cell subtype, CD8^low^CD56^+^CD3^-^CD4^-^, was observed in untreated, clinical isolated demyelination syndrome (CIS) and in relapse remitting MS (RRMS) patients, suggesting that this decrease in CD8^low ^NK cells is an early event in demyelinating diseases [24]. The action of daclizumab (anti-IL2Rα) may restore to normal levels the CD8^low^CD56^+^CD3^-^CD4^- ^subset, this expansion correlating with decreased brain inflammation and decreased survival of activated T cells [21]. Two broad subsets of NK cells have been characterised, CD56^bright ^CD16^dim/neg ^and CD56^dim ^CD16^bright/pos^, the former being more regulatory (or at least, cytokine secreting) and the latter being more cytotoxic [25]. Lunemann and colleagues found interferon-γ release from the CD56^bright ^CD16^dim/neg ^subset to be diminished in MS patients [26].

We here aimed to determine whether abnormal NCR1 expression could be found in MS patients and whether NK cells are present in white matter lesions, using the NCR1 receptor as an NK cell marker.

## Materials and methods

### Blood donors and PBMC preparation

Healthy controls were recruited within Imperial College London, while MS donors were recruited during the MS Clinic in Charing Cross, NHS Trust-Imperial College London. We recruited 9 healthy controls, 8 RRMS naive patients (i.e. no prior treatment received), 8 progressive MS (PMS) and 9 interferon-β-treated RRMS patients. Blood samples were collected in heparin tubes and processed within five hours. All donors gave informed consent previously approved by the research ethics committee (05/MRE12/8). Peripheral blood mononuclear cells (PBMCs) were isolated using Histopaque 1077 (Sigma-Aldrich, Gillingham, UK) gradients. We used freshly separated cells for immunophenotyping experiments by flow cytometry. For RNA extraction, cryopreserved PBMCs were used and treated as described in the following paragraphs.

### Flow cytometry

Single cell analysis of PBMC was carried out using multi-parameter flow cytometry. Mouse anti human CD3-Allophycocyanin (APC)-H7 (clone SK7), CD16-Fluorescein isothiocyanate (FITC) (clone B73.1), CD56-phycoerythrin (PE)-Cy7 (clone B159), NKp46- phycoerythrin (PE) (Clone 9E2/NKp46) were purchased from BD Bioscience (Becton Dickinson, Oxford, UK). Freshly isolated PBMCs were blocked on ice with 10% human serum in washing buffer (1% BSA in PBS), and subsequently stained with the cocktail of antibodies as described above for 45 min on ice, in the dark. After incubation, cells were washed twice with washing buffer (1% BSA in PBS) and fixed using a solution of 1% PFA in PBS. For each donor, we included isotype controls for CD16, CD56 and NKp46. Cell surface expression level of NKp46 is expressed as ratio of the MFI (Mean Fluorescence Intensity) of NKp46 positive cells and the MFI of matching isotype control for the same donor.

### Human tissue samples

MS and control brain tissue samples were kindly donated from the UK Multiple Sclerosis Tissue Bank (Centre for Neuroscience, Imperial College Faculty of Medicine, Hammersmith Campus, London, UK) and the human brain tissue bank in Budapest (Department of Anatomy, Semmelweis University, Budapest, Hungary). Fully informed consent and ethical approval were obtained for the collection and study of post-mortem tissue following local and guidelines recently published by the BrainNet Europe Brain Bank Consortium [27]. All post-mortem MS tissues were obtained via a UK prospective donor scheme with full ethical approval (08/MRE09/31). Neuropathological confirmation of the diagnosis of MS was carried out according to the International Classification of Diseases of the Nervous System criteria http://www.ICDNS.org. Samples were taken from 12 MS patients of which 11 were female and 1 male with ages at death ranging from 34 to 59 years (mean = 46.6). The majority of MS cases were secondary progressive (SPMS) and disease duration ranged from 2 to 36 years (mean = 16.7). The 10 control patients, 7 females and 3 males, were free of any evidence of known neurological disease, and had an average age at death of 47 (range 26-60). Further details of control and MS cases can be found in supplementary Tables 1 and 2 respectively (Additional file 1). Groups were matched for gender and age at death and when compared were not statistically different based on gender (Fisher's exact test; p = 0.2932) or age at death (*t *= 0.91) and showed similar homogeneity of variances for both group (FTest = 0.33). Lesioned tissue of MS patients was identified on serial sections by standard immunostaining for myelin oligodendrocyte glycoprotein (MOG) expression and by Luxol^® ^fast blue (LFB) solution (Sigma-Aldrich Company Ltd, UK). Parkinson's disease (PD) tissue was donated from the UK Parkinson's disease Tissue bank (Centre for Neuroscience, Imperial College Faculty of Medicine, Hammersmith Campus, London, UK). Appendix and tonsil inflamed tissue was kindly donated from the Human Biomaterials Resource Centre (Hammersmith Hospitals NHS Trust, Hammersmith Hospital, London, UK). The tonsil donor was a 19 year old female who underwent tonsillectomy due to reactive lymphoid hyperplasia and the appendix donor was female, 50 years, who underwent appendectomy.

### Cell lines and cell culture experiments

The human U251 astrocytoma cell line was a kind gift from Dr Amin Hajitou, (Department of Gene Therapy, Division of Neuroscience, Imperial College London). The cells were used in our experiments between the 3^rd ^and the 10^th ^passage. For both RNA extraction and flow cytometry, cells were plated at 0.5 × 10^6^/well or 2 × 10^6^/well. For confocal microscopy, cells were plated directly in glass bottom slides at the same cell density, having taken into account the number of cells/cm^2 ^in both culture conditions. The cells were let to adhere overnight and the following day, were divided in two groups: one was left 24 hr in DMEM without FCS, the other group in DMEM culture media supplemented with FCS. After 24 hr both groups were cultured for 72 hr with DMEM with FCS. At this time point, cells were harvested for RNA extraction or stained directly on glass slides for confocal microscopy.

For RNA extraction, cells were washed twice in cold PBS and the pellet frozen at -80°C. RT-PCR products were visualised by agarose gel electrophoresis on 2% agarose TAE gels with SYBR^® ^Safe DNA gel stain (Invitrogen, Paisley, UK). HyperLadder™ II (Bioline, London, UK) was used as the molecular weight marker. Gels were visualised with a BioDoc-It^® ^Imaging System (UVP, Cambridge, UK) and LabWorks™ image capture and analysis software. ImageJ software [28] was used to conduct as semi-quantitative analysis of expression. Mean voxel intensity of bands was used to determine product expression of the reference gene and gene of interest (GOI). GOI intensities were divided by their respective reference gene intensities to determine final expression of NCR1 (as a percentage).

For confocal microscopy, U251 cells were grown directly in cell-culture pre-treated 8- well chamber slides (LabTek™, Nunc, Thermo Fisher Scientific). At chosen time points, the chambers were removed from the slides. The slides were washed twice in ice-cold PBS, then fixed with ice-cold methanol for 30 min, air-dried and stained with NKp46 antibody or isotype control. After 45 min incubation, slides were washed twice, then incubated with donkey anti-mouse secondary antibody Alexa Fluor-488 conjugated and donkey anti-rabbit secondary antibody Alexa Fluor-680 conjugated (Invitrogen, Paisley, UK). After 45 min incubation the slides were washed twice, incubated with DAPI (2.5 ng/μL final concentration from Invitrogen, Paisley, UK) for 10 min, washed twice in PBS and once in distilled water before mounting with fluorescent mounting medium (DAKO, Ely, UK). The mounted slides were stored in the dark at 4°C.

### RNA extraction

Total RNA was extracted from dissected snap-frozen tissue (< 100 mg) according to an optimised protocol [29] using the RNeasy^® ^tissue lipid mini kit (Qiagen Ltd, Crawley, UK) according to the manufacturer's instructions, and was stored at -80°C until further use. RNA concentration and purity was assessed by spectrophotometry (NanoDrop ND1000; NanoDrop Technologies, Delaware, USA).

### Quantitative Real Time Polymerase Chain Reaction (RT-qPCR)

The two-step real-time reverse transcriptase quantitative polymerase chain reaction (RT-qPCR) was performed using the QuantiTect^® ^reverse transcription kit and the Brillant^® ^II QPCR master mix with low ROX from Agilent technologies (Agilent technologies UK Ltd, Edinburgh, UK). For cDNA synthesis, 1 μg of total RNA from each sample was reverse transcribed according to the manufacturer's instructions using the QuantiTect^® ^reverse transcription kit with integrated removal of genomic DNA contamination. No reverse-transcriptase reactions (No RT) consisted of the same protocol as above but the Quantiscript reverse transcriptase was omitted and replaced with RNase free-water. The reactions were stored at -20°C until further use. Real-time PCR experiments were performed using the Mx3000P™ real-time PCR system with software version 4.10 (Stratagene, La Jolla, USA).

For each sample, 20 μl reactions were set up in duplicate and in duplex, with each reaction containing 10 μl of 2× master mix, 2 μl of 10× primetime assay (1 μl of GOI + 1 μl of normaliser), 7 μl of RNase-free water and 1 μl template cDNA. PrimeTime™ qPCR assays were purchased from Integrated DNA technology (Coralville, Iowa, USA) and are listed in table 1. Reactions were carried out with the following cycling protocol: 95°C for 10 min, then 45 cycles with a 3-step program (95°C for 15 s, 50°C for 30 s and 72°C for 30 s). Fluorescence data collection was performed during the annealing step. Control No RT reactions to test for contaminating DNA and a negative control containing no cDNA template were introduced in each run.

**Table 1:** Primers and probes

GAPDH	Assay ID	Hs.PT.42.1164609
	Probe	5'-/5HEX/TGC GGT CAC CAT CAATGA AGA GCA/3IABkFQ/-3'
	Primer 1	5'-CGC AAT CAT AGG ACT AGA GAC G-3'
	Primer 2	5'-GAT CCT GTATTC GGCTTC CAG-3'
**NCR1**	Assay ID	Hs.PT.1994249
	Probe	5'-/56-FAM/CGAGAGGGT/ZEN/GGGTGTGTCATACATTTC/3IABkFQ/-3'
	Primer 1	5'- TCTAGACGGCAGTAGAAGGTC -3'
	Primer 2	5'- CTTGCTGGATCTGGTGGTAA -3'

**XPNPEP1**	Assay ID	Hs.PT.42.500129
	Probe	5'-/5HEX/TGCGGTCACCATCAATGAAGAGCA/3IABkFQ/-3'
	Primer 1	5'- CGCAATCATAGGACTAGAGACG -3'
	Primer 2	5'- GATCCTGTATTCGGCTTCCAG -3'

Efficiencies of the primer/probe assays were tested individually and in duplex. Expression levels of target genes were normalised to the levels of the novel XPNPEP1 [X-prolyl aminopeptidase (aminopeptidase P) 1] reference gene (unpublished data by Pascal F Durrenberger) and calibrated utilising a standard curve method for quantitation. Some experiments were then duplicated using a more commonly used normaliser; GAPDH. The calibrator was generated by creating a pool from all the control cDNA samples. Levels of the calibrator represented the baseline (of one) from which all RNA expression values were calculated within an experiment. The standard curve was used to determine relative quantity expression values for each target gene after RT-qPCR analysis of each test specimen. Relative expression values for each target gene are expressed as a ratio of target gene expression level to the reference gene expression level in the same specimen.

### Immunohistochemistry and immunofluorescence

Snap frozen tissue sections were fixed in 4% paraformaldehyde (PFA) for 10 min at 4°C and rinsed in PBS. Sections were then permeabilised and endogenous peroxidase activity removed by incubation in methanol containing 1% hydrogen peroxide for 15 min at -20°C. After two rinses, primary antibodies (Abs) was applied at tested or manufacturers recommended dilution at 200 μl per slide and left to incubate overnight at room temperature (RT). All primary antibodies are listed in Table 2. After an overnight incubation, sections were rinsed with PBS and biotinylated secondary Abs were applied at the following dilutions: anti rabbit at 1:200, anti-mouse and goat at 1:100, at 200 μl per slide and incubated for 45 mins (RT). Slides were rinsed in PBS. A complex of avidin and biotin (ABC) solution was prepared 30 min before application. Solution A and B were added at 5 μl, to 1 ml of 1% bovine serum albumin (BSA) diluted in PBS. ABC solution was applied to slides at 200 μl per slide and incubated for 60 min (RT). Slides were then rinsed in PBS. NovaRed™ (Vector^®^, Burlingame, CA, U.S.A) chromogen was prepared to manufacturer's instructions. Chromogen was applied for 1-5 min depending on the primary antibody. The reaction was stopped by rinsing in distilled water. Sections were counterstained in Mayer's Haemalum for 2-5 sec and rinsed for 5 min. Sections were dehydrated and placed in 70% methylated spirit for 1 min, then 90% for 1 min, then 100% for 1 min, and again at 100% for 1 min. Slides were then placed in xylene for 4 min and mounted with a coverslip using DPX mountant for microscopy and slides were left to dry before viewing. Immunofluorescence staining was as described for immunohistochemistry (up to the step of ABC application) except that here the secondary Abs used were fluorescently labelled and used at 1:500 (Invitrogen, Paisley, UK). After 45 min (RT) in the dark, the sections were rinsed in PBS. Coverslips were applied using fluorescent mounting medium (DAKO, Ely, UK) and DAPI (Invitrogen, Paisley, UK) and the slides dried under pressure. Reagents were purchased from VWR (UK) unless otherwise specified.

**Table 2:** Primary Antibodies

Antigen	Target	Donor Species	Working Dilution	Source
**NCR1**	Extracellular	Mouse monoclonal	1:1,500	R&D Systems, Abingdon, UK
**NCR1**	Extracellular	Goat polyclonal	1:50	R&D Systems, Abingdon, UK
**NCR1**	Full length	Mouse monoclonal	1:100	Abcam, Cambridge, UK
**NCR1**	C-terminus	Goat polyclonal	1:75	Santa Cruz Biotechnology^®^, Santa Cruz, CA, USA
**GFAP**	Astrocytes	Rabbit monoclonal	1:200	Dako UK Ltd, Ely, UK
**MOG**	Oligodendrocytes (myelin)	Mouse monoclonal	1:50	Gift of Dr S.Piddlesden, Cardiff, UK

Images were ascertained using ImagePro7 software (MediaCybernetics, Inc, Bethesda, MD, USA) and captured with a Nikon Eclipse E1000M microscope/digital camera system. Positive immunostaining was highlighted by setting the gray-level detection limits to threshold using ImageJ software [28] and the area of highlighted immunoreactivity was obtained as percentage area of the field scanned. Five fields per tissue section were scanned and the mean values were used in subsequent statistical analysis. Fluorescence images were ascertained by fluorescent microscopy using the same Nikon Eclipse E1000M microscope/digital camera system (QImaging) and the Leica TCS STED confocal microscope (Leica Microsystems GmbH, Wetzlar, Germany). Digitised images were processed using Image ProPlus (Media Cybernetics) and ImageJ and prepared in Adobe Photoshop for publication.

### Statistical Analysis

Group difference was established by using the non-parametric Mann Whitney test or parametric t test or with a one-way ANOVA with Bonferroni's multiple comparison test for multiple group comparison. Homogeneity of variance was assessed using F-test. Microsoft Office Excel 2010 (Microsoft UK Headquaters, Reading, UK) and GraphPad Prism 5.01 (GraphPad Software, La Jolla, CA) software packages were used for statistical analysis. Data shown as mean ± standard error of the mean (SEM) unless stated, differences were taken as significant when *p *< 0.05.

## Results

### NCR1 receptor expression in peripheral blood

**Figure 1:**
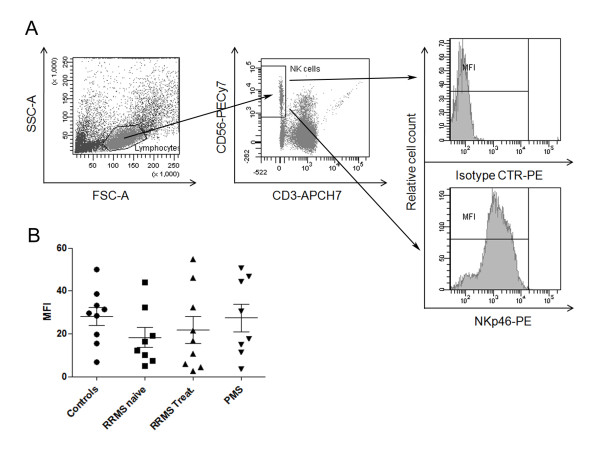
**NCR1 expression in PBMC**. (A) Representative panel indicating the gating strategy used to evaluate the expression of NCR1 on NK cells. First, lymphocytes were gated according to SSC vs FSC, then on NK cells defined as CD56^+^CD3^- ^cells. NCR1 expression was evaluated on NK cells. The top plot shows a histogram for the isotype control and the bottom plot for the NCR1 stained cells. The expression of NCR1 was calculated as the ratio between the MFI of NK cell NCR1 expression and the isotype control for each sample. (B) Graph shows the MFI ratios calculated as described above for healthy controls, RRMS-untreated, RRMS-treated and PMS donors. A one-way ANOVA was conducted with Bonferroni's correction for multiple comparison tests and no significant difference was found (lower panel). There is a trend to lower expression in naive MS patients (MFI = 18.29 ± 4.734; p = 0.07) compared to controls (MFI = 28.10 ± 4.2761) which may have reach baseline levels after treatment (MFI = 21.70 ± 6.313).

NCR1 mRNA expression levels were not significantly different between MS (n = 12) and control (n = 6; data not shown). We further used flow cytometry to compare NCR1 expression on peripheral blood NK cells of MS patients and controls. We compared the mean NCR1 fluorescence (MFI) of gated CD56^+^CD3^- ^cells from RRMS (n = 8), treated RRMS (n = 9) and progressive MS (n = 8) patients with controls (n = 9). A one-way ANOVA was conducted with Bonferroni's multiple comparison tests and no significant difference was found in expression of NCR1 between groups (and nor was there a difference in NK cell number between groups; Figure 1). There was a trend to lower NCR1 expression in MS patients (MFI = 18.29 ± 4.734; p = 0.07) than controls (MFI = 28.10 ± 4.2761) rising slightly after interferon-β treatment (MFI = 21.70 ± 6.31).

### NCR1 transcription in MS white matter lesions

**Figure 2:**
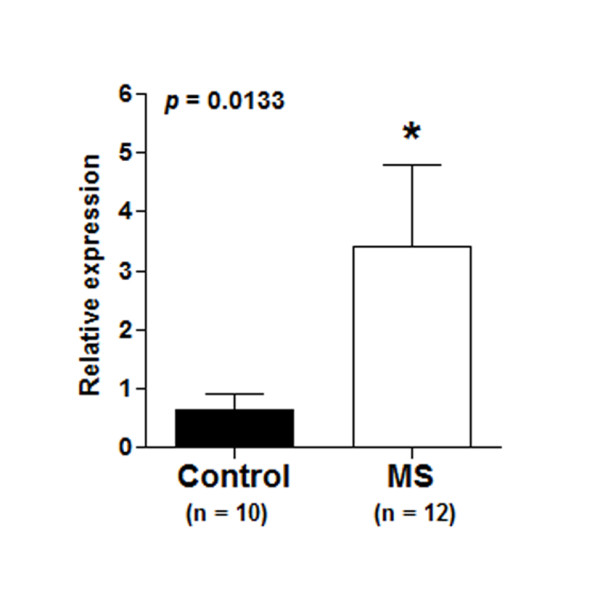
**NCR1 mRNA expression in white matter lesions**. NCR1 mRNA was investigated in whole tissue RNA extract from MS white matter lesions (WML) and control white matter. We compared levels of NCR1 mRNA and conducted a Mann Whitney test. NCR1 mRNA levels from MS WML were significantly (p = 0.0133) increased (3.418 ± 1.368) compared to controls (0.651 ± 0.246). XPNPEP1 were used as normaliser. Similar increased levels were confirmed with the more commonly used normaliser GAPDH (data not shown).

We further investigated NCR1 by looking at NCR1 mRNA levels in CNS white matter lesions (WML) from MS patients. We found a significant increase (*p *= 0.0133) in NCR1 mRNA levels in MS WML compared to controls (Figure 2). Very low to undetectable levels of NCR1 mRNA were measured in post-mortem control tissue. In MS WML, NCR1 mRNA expression varied from control levels in some cases to very high levels, particularly in cases with high levels of organised meningeal inflammation and associated substantial sub-pial grey matter and focal white matter demyelinating lesions, the latter of which contained macrophages with myelin debris indicative of active lesion formation. These MS cases have previously been characterised and reported by us [30] and were associated with earlier disease onset, irreversible disability and death [31]. NCR1 mRNA in WML was increased more than 5 times in response to disease activity (Figure 2). To verify the effectiveness of macro-dissected lesioned tissue, we verified expression levels of myelin basic protein (MBP) and myelin-associated oligodendrocytic basic protein (MOBP), which were significantly down-regulated (data not shown).

### Immunohistochemical localization of CNS NCR1 expression

**Figure 3:**
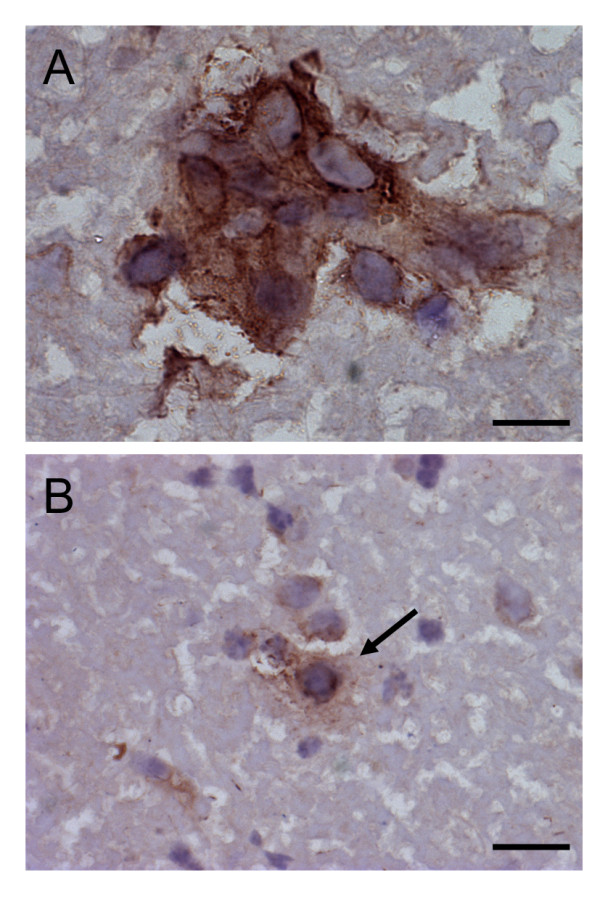
**NK cells in white matter lesions in secondary progressive MS patients**. Few NCR1^+ ^lymphocytes were detected in the CNS of MS patients, and only in cases showing active demyelination (2 cases) and presenting more active than chronic lesions. The NCR1^+ ^cells detected were found near blood vessels (A) but also in tissue (B). Scale bar = 10 μm.

Using immunohistochemical (IHC) staining we investigated *in situ *NCR1 protein expression in the same WML with mouse monoclonal anti-human NCR1 antibodies directed toward the extracellular domain of NCR1. Very few NCR1^+ ^lymphoid cells were detected in grey and white demyelinated areas and only in the cases showing extreme focal demyelination (2 cases) and presenting more active than chronic lesions. The NCR1^+ ^lymphoid cells detected were found in perivascular cuffs (Figure 3A) but also a few in tissue (Figure 3B). Using the same antibodies, numerous NCR1^+ ^NK cells were detected in inflamed appendix and tonsil as positive control tissues (Additional file 2, Page 1). Our evidence thus indicates the presence of NK cells in small numbers and only in more active lesions.

### NCR1 expression in astrocytes

**Figure 4:**
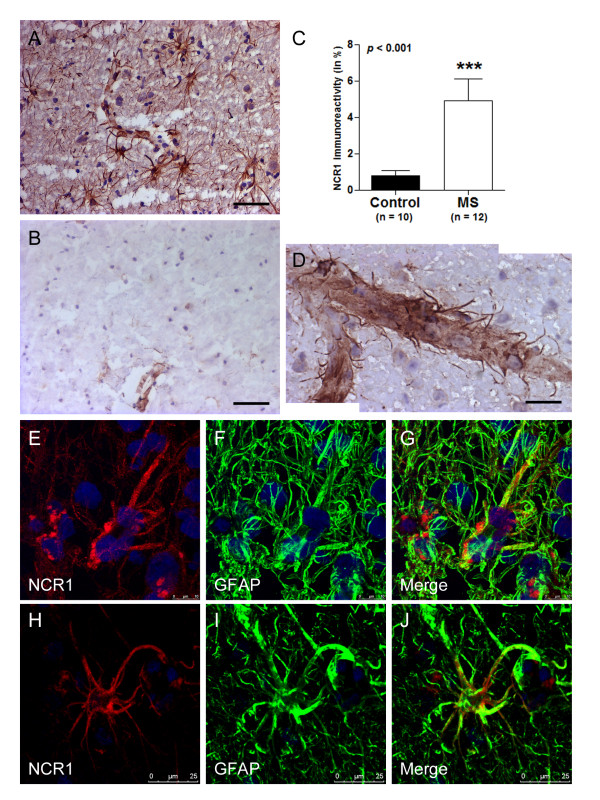
**NCR1 expression on astrocytes in WML**. In the CNS, NCR1 was expressed on astrocytes and more so in WML from MS patients (A) than in controls (B). Immunoreactivity was quantified and a significant increase (*p *= 0.0003) in WML (4.945 ± 1.174) compared to controls (0.801 ± 0.27) was found when conducting a Mann Whitney test (C). NCR1 immunoreactivity was in general concentrated on reactive astrocytes near blood vessels and in some instances, NCR1 positive end-feet could be distinctly observed (D). In addition to stellate morphology, the phenotype of NCR1^+ ^cells was confirmed using double immunofluorescence with a classical astrocyte marker, GFAP and with the mouse monoclonal to extracellular domain (E-G) and to full length NCR1 (H-J). Scale bar = 25 μm (A & B), Scale bar = 10 μm (D).

Nevertheless, the few NK cells detected in some cases could not account for the substantial up-regulation of mRNA identified by us. NCR1 has previously been identified as a definitive marker of NK cells [2]. This is the first time to our knowledge that NCR1 antibodies have been used to stain human MS brain tissue and we detected another cell type positive expressing NCR1, astrocytes. Numerous astrocytes positive for NCR1 could be observed in WML lesions from MS patients (Figure 4A) with little or no expression in control white matter (Figure 4B). In addition, tips of astrocytic processes (end-feet) terminating on blood vessel walls were also NCR1^+ ^(Figure 4D).

### Analysis of astrocyte staining with NCR1 antibodies of distinct epitope specificities

**Figure 5:**
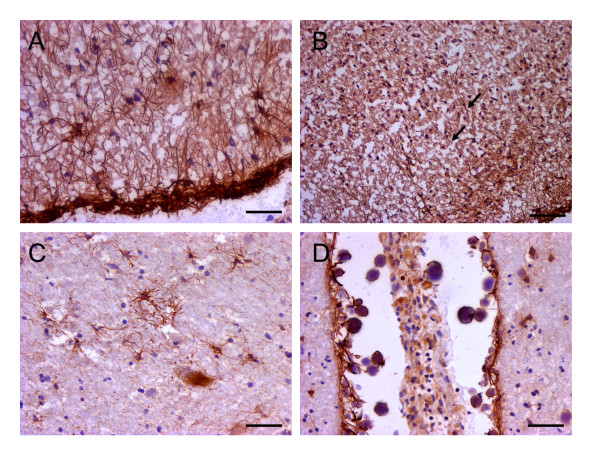
**NCR1 expression in MS grey matter and in the parkinsonian brain**. In the MS grey matter NCR1^+ ^astrocytes were observed mostly restricted to the pia surface (A) and throughout, irrespective of the presence of areas of demyelination or not. In some cases however some intra-laminar NCR1^+ ^astrocytes (B) were seen but very few NCR1^+ ^astrocytes were detected in neuronal cortical layers. NCR1^+ ^astrocytes were detected in the PD substantia nigra (B) and also in the frontal cortex (pia surface) of the parkinsonian brain (C). Scale = 20 μm (A, C & D), Scale = 40 μm (B).

To confirm the detection and specificity of expression of a novel innate receptor on astrocytes we tested 3 other NCR1 antibodies raised towards various domains of the NCR1 protein on positive control tissue (tonsils) and on human brain tissue sections. To this end we used a mouse monoclonal raised to the recombinant full length human NCR1 (Abcam), a goat polyclonal raised towards the extracellular domain (R&D Systems) and a goat polyclonal to the c-terminus (SantaCruz). All three antibodies detected positive control staining of NK cells in tonsils. Furthermore, all three antibodies stained astrocytes in human brain tissue and more so in WML than in controls. Expression levels differences between disease and control were similar for all three antibodies (Additional file 2, Page 2). We also stained tissue from grey matter lesions in the MS brain (Figure 5A &5B) and another neurodegenerative disease Parkinson's disease (PD) characterised by a loss of dopaminergic neurons in the substantia nigra, and presence of reactive astrocytosis (Figure 5C &5D). We again observed the presence of NCR1+ astrocytes.

We quantified NCR1 protein expression in white matter lesions and corroborated increases as seen at the mRNA level. NCR1 protein levels were significantly up-regulated (*p *< 0.001) in MS (4.945 ± 1.171) compared to controls (0.801 ± 0.270; Figure 4C). NCR1 positive staining was restricted to white matter lesions defined by MOG and LFB staining on serial sections. Astrocytes that were NCR1^+ ^were restricted to WML (or the pia surface in grey matter lesions) and near blood vessels (end-feet). In addition to the identification by their distinctive stellate morphology, we confirmed the astrocyte phenotype by co-localising staining with the NCR1 extracellular domain antibody (Figure 4E-G) and with the NCR1 full length antibody (Figure 4H-J) with an antibody to the astrocytic marker, GFAP, confirming expression of NCR1 on astrocytes.

### NCR1 expression in the corpora amylacea

**Figure 6:**
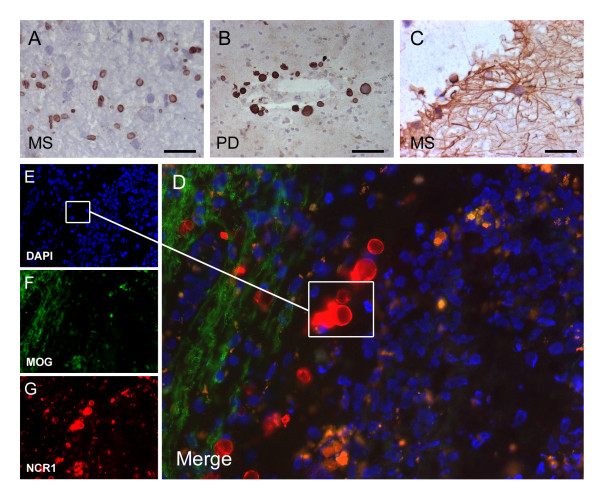
**NCR1^+ ^Corpora amylacea**. Using a goat polyclonal to the NCR1 C-terminus we detected numerous NCR1 round structures in WMLs (A). In Parkinson's disease tissue, near blood vessels we detected round structures bigger this time and more reminiscent of typical corpora amylacea (B). Using fluorescence microscopy these structures contained no nucleus confirming presence of NCR1 C-terminus in the membrane of corpora amylacea (D-G). NCR1 mouse monoclonal to extracellular domain was also able to detect only some corpora amylacea but not to the extent as the goat polyclonal to C-terminus and only at proximity to NCR1^+ ^astrocytes (C). Scale bar = 10 μm (A and D), scale bar = 25 μm (B and C).

Antibodies raised towards the C-terminus detected positivity also in the corpora amylacea. These are spherical bodies associated with neurodegeneration and containing neuronal biowaste endocytosed and packaged by astrocytes; this observation potentially links expression of the NCR1 receptor on astrocytes to the specific function of endocytosis of redundant extracellular material. The goat polyclonal to the NCR1 C-terminus detected numerous, small, cell-like spherical bodies in WML (Figure 6A). In Parkinson's disease the structures detected by this antibody were larger and more clearly reminiscent of corpora amylacea (Figure 6B). Fluorescent microscopy confirmed that these structures were un-nucleated (Figure 6D-G). The mouse monoclonal to extracellular domain of NCR1 also detected similar structures, though not to the same extent as the C-terminus specific NCR1 antibody and only in close proximity to NCR1^+ ^astrocytes, suggesting a role in the process of formation of newly formed corpora amylacea (Figure 6C and 5D).

### NCR1 expression in U251 astrocytoma cells

**Figure 7:**
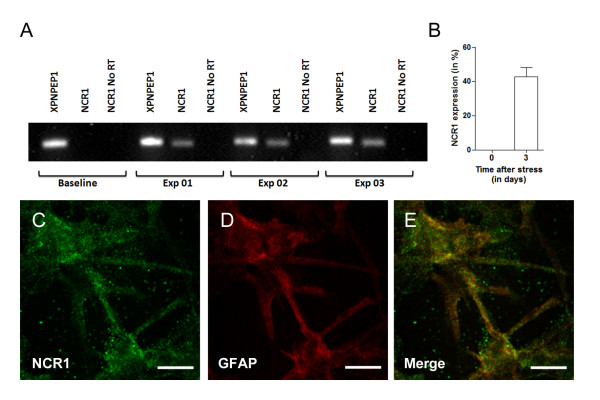
**De novo expression of NCR1 mRNA in human U251 astrocytoma cells after stress**. The human U251 astrocytoma cell line was used to look for *de novo *expression of NCR1 in astrocytes. Expression of NCR1 mRNA was detected when cells were cultures at a concentration of 2 × 10^6^/well (A). No reverse transcription samples were negative, confirming that the bands were from *de novo *NCR1 mRNA production after stress and not from genomic DNA. Semi-quantitative analysis of NCR1 expression showed a 43% increase in NCR1 expression after 3 days of culture. Double immunofluorescence (GFAP-NCR1) confirmed presence of NCR1 protein on the human U251 astrocytoma cell line (C-E).

In order to further validate *de novo *expression of NCR1 in astrocytes, we used *in vitro *analysis of human astrocytoma U251 cells, a well-characterised permanent astrocytoma cell lines derived from patients with malignant astrocytomas [32] and previously used in MS studies [17]. U251 cells were grown, alongside standard condition, in various culture conditions (cell density and media) in order to verify whether cell stress could induce expression of NCR1. We detected increasing NCR1 expression with increasing cell density. This experiment was repeated 3 times and semi-quantitative RT-qPCR of NCR1 expression was conducted (Figure 7A). Mean signal intensity (range 0-255) was measured from both bands and a percentage of NCR1 expression was established from the reference gene band (XPNPEP1). After 3 days incubation following cellular stress we detected a *de novo *increase of 43% compared to baseline (Figure 7B). We then cultured U251 cells on chamber slides and stained for NCR1 and GFAP. NCR1 expression appeared largely cytoplasmic. We also observed NCR1 expression on the cell surface of processes (Figure 7C-E).

## Discussion

We here show expression of a novel immunoglobulin superfamily member, NCR1, on astrocytes which is up-regulated in MS lesions. NCR1, which is an activating receptor of NK cells and often considered the definitive NK cell marker, was here used to investigate the presence of NK cells in MS brain and peripheral blood. Detectable changes in expression of NCR1 were restricted to the central nervous system. No significant difference in PBMC cell surface NCR1 protein expression (or mRNA) was found between MS patients and controls.

We demonstrated increased levels of mRNA and protein levels of NCR1 in white matter lesions from patients with secondary progressive MS. However, we were only rarely able to co-localize NCR1 expression to infiltrating cells of lymphoid morphology and co-expressing NK cell markers. Rather, we found the NCR1 receptor to be mainly expressed on astrocytes and on corpora amylacea. Very few NCR1^+ ^NK cells were found in the MS brain parenchyma and only in those cases showing active demyelination. Post-mortem MS brains used in this study are progressive MS patients, so that lesions were found at different stages of formation, including active lesions. Our results suggest that NK cells, being part of the innate response, may be restricted to early lesion formation as no NCR1^+ ^NK cells were detected in chronic active lesions. All in all, we show that NCR1 is not exclusive to NK cells and is expressed on reactive astrocytes, suggesting an active role of this molecule on astrocytes. Such a role may be in phagocytosis and the control of redundant extracellular material. Another possible role is suggested by analogy to the function of another NK cell receptor family member, LILRB1, which is expressed by dendritic cells [33]. There it appears to serve a functional role since cell-surface ligation leads to changes in differentiation and cytokine release [33,34].

NCR1, once considered a marker solely of NK cells, has been identified on lymphocyte subsets other than NK cells. Malignant CD4^+ ^T lymphocytes from patients with Sézary syndrome, an aggressive cutaneous T-cell lymphoma, express NCR1 mRNA and protein at the cell surface where it appears to display a novel inhibitory function [35]. Another study identified a small subset of NCR1^+ ^natural killer T cells (NKT) cells that in the presence of interleukin-15 (IL-15) are susceptible to leukemic transformation and become a functionally distinct subset [36].

NK cells are classified as part of a family of haematopoietic effectors cells referred to as innate lymphoid cells (ILCs). These ILCs have a principal protective function in response to infectious agents. Other functions attributed to these cells are assistance in lymphoid tissue formation, in tissue remodelling after injury and in homeostasis of tissue stromal cells [37]. Astrocytes are able to produce cytokines such as IL-1β, TGF-β, IL-4, IL-6, IL-10, IL-12, IL-17, IL-23 [38] and more recently IL-15 [39], altering the inflammatory cytokine milieu for adaptive immunity. Their role thus extends beyond support for neurones to an active role as regulators in CNS immunity. The current view on astrocyte origin suggests that they derive from radial glia emerging from the neural crest [40]. This is thus a completely distinct lineage to the ILCs, here found to express NCR1. Nevertheless other IgSF members have been found on astrocytes. In 2003, a Japanese team isolated a novel dual Ig domain (V-type) immunoglobulin, limitrin (also known as matrix-remodelling associated 8), on astrocytic end-feet and based on this evidence suggested that limitrin is physically and functionally associated with the blood-brain barrier [41]. Many members of the immunoglobin superfamily mediate either homophilic or heterophilic cell adhesion interactions, and serve as receptors mediating cell to cell or cell to extracellular matrix interactions [42]. One of the most prevalent immunoglobulin cell adhesion molecule (IgCAM) in the vertebrate nervous system is N-CAM1 (neural cell adhesion molecule 1; CD56). Homophilic interactions between N-CAM molecules on adjacent cells are thought to bind cells together [43]. CD56 is widely used as a marker defining NK cells [25] though expression is also found on neurones, oligodendrocytes and astrocytes. NCAM1 or its polysialylated form (PSA-NCAM), was shown to be expressed on demyelinated neurones [44] and widespread distribution in the CNS has been extensively documented [45]. Three main isoforms with molecular weights of 120, 140, and 180 kDa are derived from the single gene. NCAM-120 is the predominant form expressed by mature oligodendrocytes [46]. PSA-NCAM was colocalised with reactive astrocytes in the rat [47] and in humans [48]. Thus astrocytes and NK cells have not just one receptor in common but, based on our evidence, a second receptor, NCR1.

In our study, staining of the NCR1 C-terminus was mostly localised to the corpora amylacea. Corpora amylacea are globular basophilic bodies between 10 to 50 μm in diameter, which are believed to develop in astrocytic processes and to be associated with neurodegeneration [49]. These round inclusions appear with normal aging after the age 40 and are rarely observed in the adolescent brain [50]. Mostly observed in Alzheimer's disease [51] and in PD [52], there are also reports of prevalence of these inclusions in MS. For instance, proteomic analysis from MS patients' lesions confirms that these bodies contained mostly neuronal aggregates with highly polymerised cytoskeletal material [53]. Whether NCR1 is involved in the formation of these inclusions remains to be fully determined.

In order to further validate our findings, we analysed NCR1 expression in U251 cells. This confirmed expression of NCR1 at the mRNA and protein level in cells grown with serum deprivation or at high density.

## Conclusions

Taken together, the data presented here indicate *de novo *expression of NCR1 receptor on astrocytes in MS white matter lesions, in addition to the more marginal population of NCR1^+ ^NK cells. Other adhesion molecules such as CD56 are also expressed by astrocytes, along with cytokines, supporting a role of astrocytes in innate immune activation in the CNS in MS. It remains to be determined whether NCR1 on astrocytes can signal or function as co-receptor. We favour the possibility that it may be acting as an innate receptor for local stress in the form of altered carbohydrate moieties. In any case, our results argue for NCR1 as a potential CNS tissue marker of neurodegenerative disease.

## Competing interests

The authors declare that they have no competing interests.

## Authors' contributions

PFD designed and carried out the experimental work, recruitment and prepared the manuscript. AE equally contributed towards experimental work, recruitment, experimental work and manuscript preparation. LVW and MS assisted with the human tissue experimental work. FK assisted with human peripheral blood experimental work and patient recruitment. RN and OM provided access to required patients and to relevant clinical data. RR, RJB and DMA helped with the design, the interpretation of the results and the preparation of the manuscript. All authors read and approved the final manuscript.

## Supplementary Material

Additional file 1 **Basic characteristics from controls and MS patients as well as basic clinical date from MS patients**. Click here for file

Additional file 2 **NCR1^+ ^cells in inflamed appendix and tonsil (1) and comparison of NCR1 antibodies (2)**. Page 1 contains the staining of NCR1 (mouse monoclonal extracellular domain) on positive control tissue (appendix and tonsil). Page 2 contains a graphic representation of NCR1 and staining of 3 other commercially available antibodies, monoclonal full length, goat polyclonal extracellular domain and goat polyclonal c-terminus, on tonsil, MS brain tissue and control tissue respectively. Click here for file
