# Supplementary material for: Innate Immunity in multiple sclerosis white matter lesions: expression of natural cytotoxicity triggering receptor 1 (NCR1)
Source: J Neuroinflammation. 2012 Jan 2;9:1. doi: 10.1186/1742-2094-9-1 (PMC3269367; doi:10.1186/1742-2094-9-1)
Supplement: Additional file 1 — Basic characteristics from controls and MS patients as well as basic clinical date from MS patients. [file 1742-2094-9-1-S1.PDF]

Additional file 1

1. Control basic characteristic

Case	Gender	Age at death (Yrs)	PMD (Hrs)	Cause of Death
**C01**	F	56	5	Myocardial infarction
**C02**	M	37	4.5	Myocardial infarction
**C03**	F	48	5	Myocardial infarction
**C04**	F	39	12	Suicide (hanging)
**C05**	F	44	5	Myocardial infarction
**C06**	M	52	2.5	Heart failure
**C07**	F	55	5	Bronchopneumonia
**C08**	F	26	6.5	Acute cardiac insufficiency
**C09**	M	53	2	Heart failure
**C10**	F	60	13	Carcinoma of the tongue
**n = 10**	**F = 7**<br>**M = 3**	**47** (26-90)	**6.1** (2-13)	

Yrs = Years, Hrs = Hours

2. MS patient basic characteristics and clinical data

MS case	Gender	Age at Death (Yrs)	Illness Duration (Yrs)	PMD (Hrs)	MS Class	Cause of Death
**MS01**	F	42	6	11	PP	Bronchopneumonia
**MS02**	F	34	11	12	SP	Pneumonia
**MS03**	F	44	16	18	SP	Aspiration Pneumonia
**MS04**	F	45	6	28	RR	Multi-organ failure from septicaemia due to UTI
**MS05**	F	52	36	7	SP	Bronchopneumonia
**MS06**	F	57	27	13	SP	Myocardial Infarct,
**MS07**	F	44	19	20	SP	UTI, sepsis
**MS08**	F	46	25	10	SP	Pneumonia
**MS09**	M	51	5	13	PP	Bronchopneumonia
**MS10**	F	59	31	21	SP	Pneumonia
**MS11**	F	35	2	9	SP	MS
**MS12**	F	50	Unknown	22	SP	Aspiration Pneumonia
**n =12**	**F = 11**<br>**M = 1**	**46.6** (34-59)	**16.7** (2-36)	**15.3** (9-28)		

PP = Primary progressive, SP = Secondary Progressive,
