# Supplementary material for: Innate Immunity in multiple sclerosis white matter lesions: expression of natural cytotoxicity triggering receptor 1 (NCR1)
Source: J Neuroinflammation. 2012 Jan 2;9:1. doi: 10.1186/1742-2094-9-1 (PMC3269367; doi:10.1186/1742-2094-9-1)
Supplement: Additional file 2 — NCR1+ cells in inflamed appendix and tonsil (1) and comparison of NCR1 antibodies (2). Page 1 contains the staining of NCR1 (mouse monoclonal extracellular domain) on positive control tissue (appendix and tonsil). Page 2 contains a graphic representation of NCR1 and staining of 3 other commercially available antibodies, monoclonal full length, goat polyclonal extracellular domain and goat polyclonal c-terminus, on tonsil, MS brain tissue and control tissue respectively. [file 1742-2094-9-1-S2.PDF]

1. NCR1+ cells in inflamed appendix and tonsil.

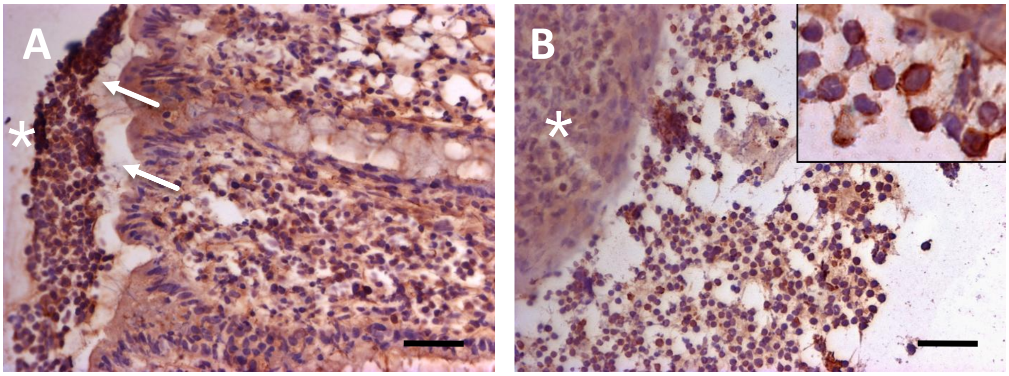

The mouse monoclonal antibodies directed towards the extracellular domain of NCR1 was tested on positive control tissue – appendix (A) and tonsil (B). In A asterisk = lumen and in B asterisk = germinal centre. Scale bar = 25 µm

2. Comparison of other antibodies.

Schematic representation of NCR1

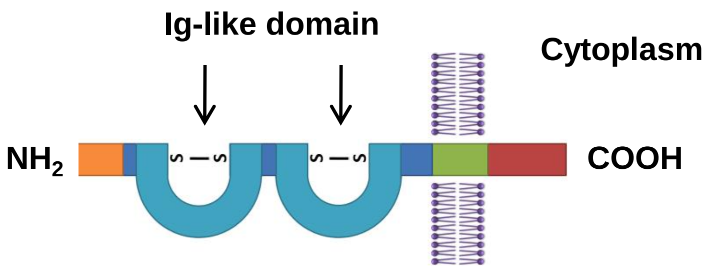

NCR1 mouse monoclonal - full length

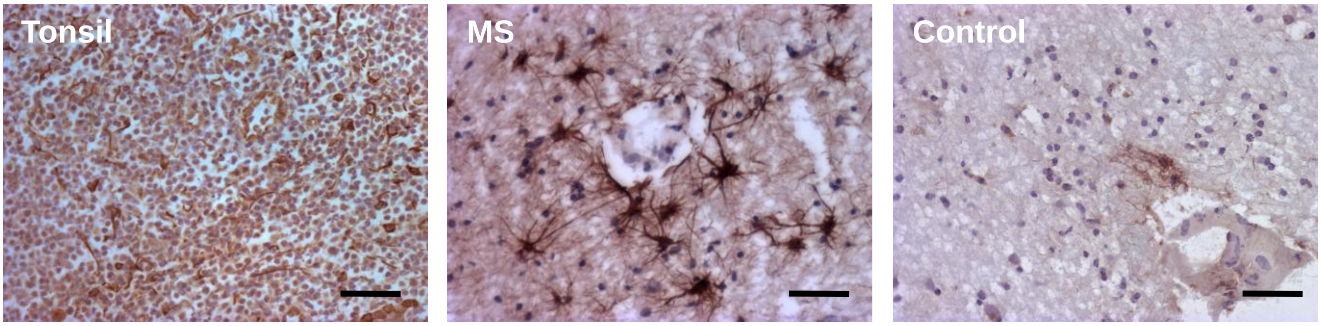

NCR1 goat polyclonal - extracellular domain

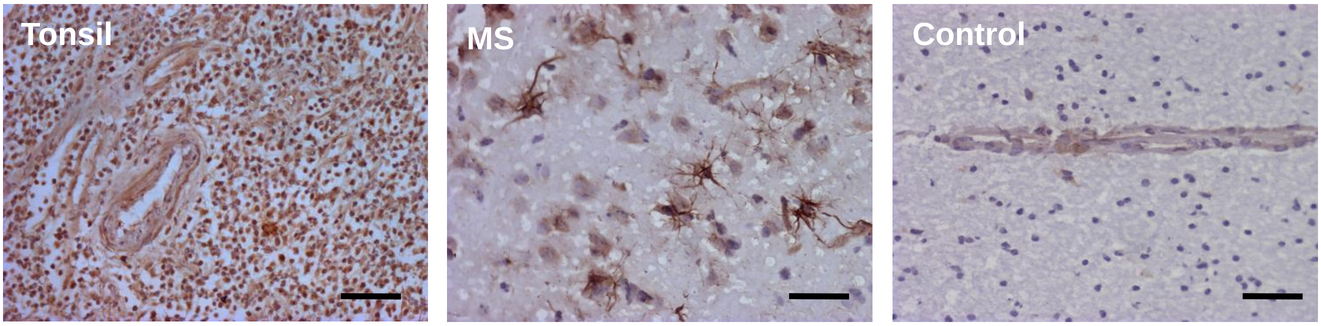

NCR1 goat polyclonal - C-terminus

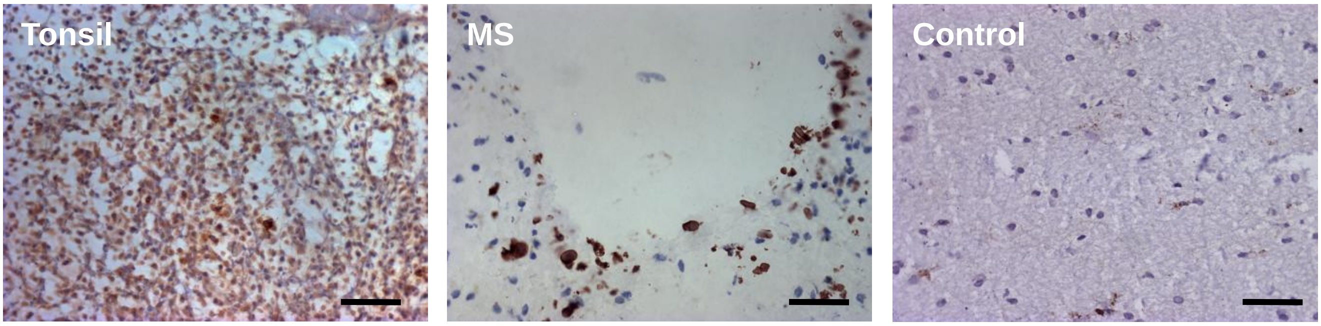

Scale bar = 25 µm
